# Mycotoxin Production in Liquid Culture and on Plants Infected with *Alternaria* spp. Isolated from Rocket and Cabbage

**DOI:** 10.3390/toxins7030743

**Published:** 2015-03-05

**Authors:** Ilenia Siciliano, Giuseppe Ortu, Giovanna Gilardi, Maria Lodovica Gullino, Angelo Garibaldi

**Affiliations:** 1 Agroinnova—Centre of Competence for the Innovation in the Agro-Environmental Sector, University of Turin, Largo Paolo Braccini 2, Grugliasco, Turin 10095, Italy; E-Mails: ilenia.siciliano@unito.it (I.S.); giovanna.gilardi@unito.it (G.G.); marialodovica.gullino@unito.it (M.L.G.); angelo.garibaldi@unito.it (A.G.); 2 DISAFA—Dipartimento di Scienze Agrarie, Forestali e Alimentari, University of Turin, Largo Paolo Braccini 2, Grugliasco, Turin 10095, Italy

**Keywords:** *Alternaria* toxins, HPLC-MS/MS, *Eruca sativa*, *Brassica oleacea*

## Abstract

Fungi belonging to the genus *Alternaria* are common pathogens of fruit and vegetables with some species able to produce secondary metabolites dangerous to human health. Twenty-eight *Alternaria* isolates from rocket and cabbage were investigated for their mycotoxin production. Five different *Alternaria* toxins were extracted from synthetic liquid media and from plant material (cabbage, cultivated rocket, cauliflower). A modified Czapek-Dox medium was used for the *in vitro* assay. Under these conditions, more than 80% of the isolates showed the ability to produce at least one mycotoxin, generally with higher levels for tenuazonic acid. However, the same isolates analyzed *in vivo* seemed to lose their ability to produce tenuazonic acid. For the other mycotoxins; alternariol, alternariol monomethyl ether, altenuene and tentoxin a good correlation between *in vitro* and *in vivo* production was observed. *In vitro* assay is a useful tool to predict the possible mycotoxin contamination under field and greenhouse conditions.

## 1. Introduction

*Alternaria* is a common, cosmopolitan fungal genus with several species pathogenic on a wide range of crops, including cereals, vegetables, fruits, ornamentals and oil-seed crops. Several *Alternaria* spp. produce leaf spots diseases in the field as well as fruit rot in the field and during transit and storage, causing serious economic losses [1]. In addition to incite plant disease, they can act as allergens, affecting immuno-compromised patients [2] and, under suitable conditions, produce powerful toxic secondary metabolites [3] with mutagenic and teratogenic potential, responsible for certain types of cancer. The main *Alternaria* mycotoxins that occur naturally are tenuazonic acid, alternariol monomethyl ether, alternariol, altenuene, and altertoxin [4]. Based on their chemical structures it is possible to divide *Alternaria* toxins into five different classes: (1) dibenzo-α-pyrones: alternariol (AOH), alternariol monomethyl ether (AME), altenuene (ALT); (2) tetramic acid derivatives: tenuazonic acid (TeA) and iso-tenuazonic acid (iso-TeA); (3) perylene quinones: altertoxins I, II and III (ATX-I, ATX-II and ATX-III) and stemphyltoxin III; (4) AAL-toxins, abbreviation for *A. alternata* f. sp. *lycopersici* toxins, including 2 groups, AAL-TA and AAL-TB. The fifth class contains miscellaneous structures such as tentoxin (TEN), a cyclic tetrapeptide. *Alternaria* toxins have been found in several agricultural commodities [1], including grains [5], sunflower seeds [6], oilseed rape, sorghum, pecans [7].

The production of *Alternaria* toxins under natural infection or as a consequence of artificial inoculation has been evaluated also in a number of fruit and vegetables, such as apple, tomato, blueberry [8], orange, lemon [9] and mandarin [10].

During the past years, several *Alternaria* leaf spots have been detected on different vegetable crops, and in most cases the pathogen resulted to be seed-transmitted. For instance, cruciferous plants are frequently damaged by *Alternaria* spp. and the pathogen is often seed transmitted [11].

In Italy, *Alternaria* spp. have been recently detected on plants and seeds of wild and cultivated rocket and basil [12,13]; in addition, they are the most common and destructive pathogens of cabbage and cauliflower. Among the known species, *A. japonica* is reported on wild and cultivated rocket [14,15], Chinese cabbage [16] and turnip [17].

The present study was carried out in order to verify the production of mycotoxins in liquid culture (*in vitro*) and on cabbage, cultivated rocket (*Eruca sativa*) and cauliflower (*Brassica oleracea*), artificially inoculated with several isolates of *Alternaria* spp. obtained from infected leaves and seeds of different hosts.

## 2. Results

A quantitative assessment of the matrix effect, as proposed by Matuszewski [18], was carried out in order to define what are critical aspects in the quantification of mycotoxins in different matrices. Two sets of samples were prepared: (i) standards of the analytes were dissolved in mobile phase; (ii) the extraction was performed from all matrices (culture medium, cabbage, cauliflower and cultivated rocket) not inoculated with the pathogen and after the analytes were added.

In Table 1 values related to matrix effect showed the higher ionization suppression for TeA, for all matrices. In order to minimize the susceptibility related to matrix effect the quantification of the analytes was obtained through the calibration curves prepared in matrices.

A third set of samples was prepared to evaluate the recovery; standards were added before extraction procedure at three concentration levels. Three replicates were prepared for each concentration and the average of the results was reported in Table 1.

**Table 1:** Validation parameters for the matrices object of study.

Validation parameters	TeA	AOH	AME	ALT	TEN
	LIQUID COLTURE				
Recovery (%) ± SD	79.9 ± 4.8	81.4 ± 8.5	86.0 ± 11.2	100 ± 5.8	79.9 ± 4.8
LOD [μg/kg]	5.79	2.31	2.20	2.31	1.34
LOQ [μg/kg]	19.3	7.69	7.35	7.69	4.45
ME (%)	27.2	47.7	34.8	74.7	182
	CAULIFLOWER				
Recovery (%) ± SD	49.0 ± 1.3	71.8 ± 1.6	102 ± 3.4	85.0 ± 4.3	51.1 ± 10.6
LOD [μg/kg]	8.62	6.90	4.60	7.91	8.24
LOQ [μg/kg]	28.7	23.0	15.3	26.4	27.5
ME (%)	16.5	52.1	58.4	67.1	44.4
	CABBAGE				
Recovery (%) ± SD	41.3 ± 3.3	83.5 ± 3.4	103 ± 5.0	93.4 ± 3.3	57.8 ± 7.5
LOD [μg/kg]	10.8	3.78	1.94	5.87	18.2
LOQ [μg/kg]	35.9	12.6	6.46	19.6	60.7
ME (%)	17.8	84.8	59.9	57.7	95.9
	CULTIVATED ROCKET				
Recovery (%) ± SD	43.0 ± 5.4	62.1 ± 2.8	73.6 ± 2.6	76.4 ± 1.1	62.1 ± 1.7
LOD [μg/kg]	13.8	6.31	6.46	5.84	1.36
LOQ [μg/kg]	46.1	21.0	21.5	19.5	4.53
ME (%)	17.2	54.9	58.4	51.9	93.5

The recovery of the five analytes in the liquid culture medium was in a range between 79.9% and 100%. The recovery for TeA (41%–49%) and TEN (51%–62%), calculated with external calibration method, was lower in vegetable matrices, and was similar to those obtained for tomato by Asam *et al.* [19,20] and Liu *et al.* [21] when they used the external calibration. However, the same authors improved their results using stable isotope dilutions.

Signal-to-noise method was used to determine limits of detection (LOD) and quantification (LOQ) for each matrix. *S*/*N* ratio 3:1 was used for the determination of LOD, while 10:1 for LOQ.

### 2.1. Quantification of Mycotoxins in Vitro

A total of 28 *Alternaria* strains were analyzed for mycotoxin production. A high percentage (>80%) of tested strains were able to produce at least one mycotoxin (Table 2).

Thirty-six percent of the isolates showed the ability to produce simultaneously four mycotoxins (TeA, AOH, AME and ALT) but not tentoxin. Another group (14%) produced all investigated mycotoxins. Seven strains (25%) did not show the capability to produce any mycotoxins. Furthermore, five strains (18%) produced only TeA and two strains (7%) produced three mycotoxins (TeA, AOH and AME) (Table 2).

**Table 2:** Profile of mycotoxins produced by *Alternaria* spp. in liquid cultures (*in vitro*).

*Alternaria* Code Number	Mycotoxin Quantification [μg/kg]				
	TeA	AOH	AME	ALT	TEN
Cav 2/10	5980 ± 791	37.8 ± 9.50	11.7 ± 0.7	161 ± 18.9	N.D. ^a^
Cav 3/10	2160 ± 16.1	13.8 ± 2.21	11.4 ± 0.3	47.2 ± 10.8	11.2 ± 1.72
Cav 5/10	7140 ± 446	2.82 ± 0.30	11.9 ± 0.5	745 ± 13.1	N.D.
Cav 7/10	63.3 ± 1.64	3.20 ± 0.31	4.20 ± 0.12	126 ± 37.5	N.D.
Cav 12/10	3210 ± 68.1	120 ± 12.9	8.87 ± 0.65	171 ± 29.5	16.0 ± 3.04
Cav 15/10	2330 ± 408	118 ± 13.5	9.73 ± 0.09	319 ± 17.3	N.D.
Ruc 1/10	1310 ± 149	42.0 ± 7.42	12.4 ± 2.11	102 ± 8.3	N.D.
Ruc 2/10	51.6 ± 17.7	1280 ± 139	851 ± 44.6	8330 ± 520	N.D.
Ruc 3/10	24.9 ± 12.6	N.D.	N.D.	N.D.	N.D.
Ruc 4/10	7240 ± 1090	90.2 ± 18.1	4.73 ± 0.58	N.D.	N.D.
Ruc 5/10	6570 ± 585	104 ± 32.1	11.9 ± 2.17	N.D.	N.D.
Ruc 6/10	1540 ± 337	N.D.	N.D.	N.D.	N.D.
Ruc 9/10	4900 ± 447	147 ± 37.6	16.8 ± 2.37	433 ± 33.7	3.13 ± 0.95
Ruc 12/10	4930 ± 578	65.9 ± 17.5	11.2 ± 1.00	292 ± 46.9	4.24 ± 1.31
Ruc 13/10	5840 ± 324	22.1 ± 0.62	19.4 ± 0.84	465 ± 14.0	N.D.
Ruc 1/11	N.D.	N.D.	N.D.	N.D.	N.D.
Ruc PMP 4	13.6 ± 3.76	N.D.	N.D.	N.D.	N.D.
Ruc PMP 8	164 ± 23.5	N.D.	N.D.	N.D.	N.D.
Ruc PMP 9	970 ± 31.1	264 ± 38.1	474 ± 21.5	30,800 ± 740	N.D.
Ruc PMP 12	3510 ± 270	72.3 ± 10.0	21.1 ± 2.08	270 ± 39.6	N.D.
Ruc PMP 19	17.0 ± 3.13	N.D.	N.D.	N.D.	N.D.
Q36-4NL	N.D.	N.D.	N.D.	N.D.	N.D.
Q37-16NL	N.D.	N.D.	N.D.	N.D.	N.D.
Q38-1NL	18.8 ± 2.82	5.69 ± 2.98	7.82 ± 0.96	1040 ± 25.5	N.D.
Q38-9NL	N.D.	N.D.	N.D.	N.D.	N.D.
Q38-19NL	N.D.	N.D.	N.D.	N.D.	N.D.
Q43-1NL	N.D.	N.D.	N.D.	N.D.	N.D.
Q43-2NL	N.D.	N.D.	N.D.	N.D.	N.D.

^a^ N.D. = not detected. Mean values ± standard error of three independent biological experiments consisting of three technical replicates each.

TeA, the main toxin produced, has been found in 75% of tested isolates and only seven strains did not show the ability to produce this mycotoxin. High variability existed referring to mycotoxin concentrations, ranging from 13.6 to 7240 μg/kg.

The benzopyrone derivatives (AOH, AME and ALT) (Table 2) were synthesized by almost the same strains. AOH and AME were produced by about 57% and ALT by 50% of the analyzed strains. On the contrary, the mycotoxin concentrations showed different trend among the same strain. In fact, AOH levels varies from 2.82 to 1280 μg/kg, AME from 4.20 to 851 μg/kg and ALT from 47.2 to 30,800 μg/kg.

Finally, TEN was detected in only 4 samples. The concentrations were very low (from 3.13 up to 16.0 μg/kg) when compared with the other mycotoxins.

### 2.2. Disease Severity

All isolates of *Alternaria* spp. artificially inoculated on cultivated rocket showed a moderate disease severity with the exception of Ruc 5\10, that showed a low value of disease severity (Table 3). Generally, the tested isolates caused only slight leaf necrosis and low virulence on both cabbage and cauliflower, with the exception of Cav 7\10 and Cav 3\10 that produce severe leaf spot symptoms on cauliflower (Table 3).

**Table 3:** Disease severity caused by isolates of *Alternaria* spp. tested for mycotoxin production.

Isolate	Host	% Affected Leaf Area	Reaction ^a^
Cav 2\10	Cauliflower	12.5	L
Cav 3\10	Cauliflower	55	H
Cav 5\10	Cauliflower	27.5	L
Cav 7\10	Cauliflower	57.5	H
Cav 12\10	Cauliflower	25	L
Cav 2\10	Cabbage	15	L
Cav 3\10	Cabbage	20	L
Cav 5\10	Cabbage	15	L
Cav 7\10	Cabbage	10	L
Cav 12\10	Cabbage	20	L
Ruc PMP 12	Cultivated rocket	34	M
Ruc PMP 19	Cultivated rocket	34	M
Ruc 3\10	Cultivated rocket	30	M
Ruc 4\10	Cultivated rocket	40	M
Ruc 5\10	Cultivated rocket	26	L
Ruc 6\10	Cultivated rocket	36	M
Ruc 12\10	Cultivated rocket	42	M
Ruc 13\10	Cultivated rocket	40	M

^a^ Reaction: NP, non-pathogenic strain; L, low virulence (10%–30%); M, moderate virulence (31%–50%); H, high virulence (51%–100%).

### 2.3. Quantification of Mycotoxin in Vivo

TeA was detected only in two samples, each one of rocket and cauliflower. Also TEN was detected only in a few samples, one of cabbage and two of cauliflowers. However, the benzopyrone derivatives (AOH, AME and ALT) were detected in some samples of rocket and in all samples of cabbage and cauliflower (Table 4).

Tenuazonic acid production *in vivo* was very low opposed to what was found in the *in vitro* studies; only one strain from rocket (Ruc 6/10) and one from cauliflower (Cav 5/10) were positive. The production of AOH, AME and ALT was similar *in vivo* (Table 4) as *in vitro* (Table 2); the strains that did not produce mycotoxins *in vitro* were negative also *in vivo*, those which were positive *in vitro* were also positive in the majority of samples tested *in vivo*. Indeed, it was possible to find a positive match for all tested isolates of cabbage and cauliflower, only one strain (Ruc PMP 12) tested on rocket gave deviating results for the production of AOH and ALT. The production of TEN was very low both *in vitro* and *in vivo*. However, two strains (Ruc 12/10 and Cav 12/10) resulted in an opposite trend.

**Table 4:** Mycotoxins produced by *Alternaria* spp. in *in vivo* samples.

Isolate	TeA	AOH	AME	ALT	TEN
CAULIFLOWER [μg/kg]					
Cav 2/10	N.D. ^a^	16.7 ± 6.77	32.11 ± 4.8	444 ± 22.0	N.D.
Cav 3/10	N.D.	2050 ± 93.6	373 ± 29.1	391 ± 47.0	20.5 ± 4.28
Cav 5/10	475 ± 56.7	94.5 ± 9.57	6.72 ± 1.71	254 ± 61.9	N.D.
Cav 7/10	N.D.	3.83 ± 0.11	593 ± 32.7	865 ± 86.7	N.D.
Cav 12/10	N.D.	10.8 ± 2.27	12.1 ± 2.09	102 ± 17.3	N.D.
CABBAGE [μg/kg]					
Cav 2/10	N.D.	8.0 ± 0.71	13.3 ± 4.0	82.8 ± 11.8	N.D.
Cav 3/10	N.D.	1850 ± 46.5	239.4 ± 42.3	661 ± 37.1	1.86 ± 0.08
Cav 5/10	N.D.	72.5 ± 13.9	8.47 ± 0.58	274 ± 27.5	N.D.
Cav 7/10	N.D.	8.29 ± 2.35	16.3 ± 1.20	470 ± 2.45	N.D.
Cav 12/10	N.D.	6.51 ± 1.51	2.26 ± 0.32	435 ± 27.4	3.98 ± 0.94
CULTIVATED ROCKET [μg/kg]					
Ruc PMP 12	N.D.	N.D.	3.60 ± 0.15	N.D.	N.D.
Ruc PMP 19	N.D.	N.D.	N.D.	N.D.	N.D.
Ruc 3/10	N.D.	N.D.	N.D.	N.D.	N.D.
Ruc 4/10	N.D.	311 ± 14.4	1280 ± 48.2	N.D.	N.D.
Ruc 5/10	N.D.	16.9 ± 2.32	4.46 ± 0.92	N.D.	N.D.
Ruc 6/10	70.0 ± 15.1	N.D.	N.D.	N.D.	N.D.
Ruc 12/10	N.D.	16.9 ± 0.77	83.4 ± 8.48	80.2 ± 12.5	N.D.
Ruc 13/10	N.D.	125 ± 2.31	293 ± 68.4	125 ± 10.3	N.D.

Mean values ± standard error of three independent biological experiments consisting of three technical replicates each. ^a^ N.D. = not detected.

## 3. Discussion

Our results show that different *Alternaria* sp. isolates from rocket and cabbage plants are able to produce five different mycotoxins under *in vitro* conditions. Most of the isolates obtained from both host plants were characterized by the production of high quantity of TeA *in vitro*. However, when tested *in vivo*, the same isolates lost their ability to produce TeA and in almost all cases it was not possible to detect this mycotoxin. Only two strains produce TeA *in vivo*: Ruc 6/10 with 70 µg/kg on cultivated rocket and Cav 5/10 but only in cauliflower with 475 µg/kg (Table 4). This is in contrast with other results obtained in previous works. In fact, TeA has been indicated as a major mycotoxin on naturally infected tomato grown in southern Italy, with levels up to 7.2 mg/g while alternariol and alternariol methyl ether were present at lower levels [22]. Also Stinson *et al.* [8,9] reported a high level of tenuazonic acid in inoculated and naturally infected tomatoes. In our experiment the different growth conditions (leaf respect fruit) seem able to influence the TeA productions.

Several isolates of *Alternaria* spp. were identified as producers of alternariol and of alternariol methyl ether, both in liquid media and in solid rice medium [23]. The mycotoxin production depended on different factors. Sanchis and Magan [24] showed that water activity (aw) played an important role for the TeA, AME and AOH production in *A. alternata* indicating an optimum value greater than 0.97. Also temperature is an important factor with an optimum at 28 °C for AOH and AME and 21 °C for TEA in synthetic medium for *A. alternata* [25]. Moreover, light exposure had a strong influence on AOH with a reduced mycotoxin production when compared to cultures grown in the dark [26,27]. Finally, Brzonkalik *et al.* [28] showed that cultivation conditions such as carbon and nitrogen source can influence mycotoxin production by *A. alternata* grown in synthetic medium. Mycotoxin production also depends on the type of plant and cultivar affected, on geographical location where the plant is grown and harvested as well as on climate [29].

For the other four mycotoxins (AOH, AME, ALT and TEN), results showed a correlation between the mycotoxin production under *in vitro* and *in vivo* conditions.

## 4. Materials and Methods

### 4.1. Isolate Collection and Propagation

Isolates of *Alternaria* spp. obtained from leaves (16 isolates) and seeds (12 isolates) of different hosts (cabbage, cauliflower, wild and cultivated rocket) were tested (Table 5 and Table 6). All *Alternaria* isolates were obtained by placing pieces of infected tissue from rocket and cabbage onto potato dextrose agar (PDA) and incubated at 25 °C for 7 days. The strains were then transferred onto potato carrot agar (PCA) amended with 0.5 mg/mL streptomycin sulphate, and incubated for 7 days at 25 °C. Single-spore cultures were established for each isolate by serial dilution of conidial suspension; a drop of 10^−6^ and 10^−8^ dilutions was plated on PDA medium. A single germinated macroconidia ware selected under stereomicroscope and transferred onto a new PDA plate. The tested strains of *Alternaria* spp. from brassica plants and seeds were identified by morphological observations as well as by a phylogenetic analysis based on β-tubulin gene and anonymous region OPA10-2 sequences [14].

**Table 5:** List of the isolates of *Alternaria* spp. obtained from leaves of different hosts in northern Italy.

Isolate Code	Species	Host	Origin
Cav 2/10	*A.alternata* complex	Cauliflower cv. White excell	Boves (CN)
Cav 3/10	*A.alternata* complex	Cabbage cv. Morama	Moncalieri (TO)
Cav 5/10	*A. arborescens*	Cabbage cv.Estoryl	Asti (AT)
Cav 7/10	*A.alternata* complex	Cabbage cv. Morama	Savigliano (CN)
Cav 12/10	*A.alternata* complex	Cauliflower cv. White excell	Boves (CN)
Cav 15/10	*A.alternata* complex	Cabbage cv. Dama	Boves (CN)
Ruc 1/10	*A.alternata* complex	Cultivated rocket	Moncalieri (TO)
Ruc 2/10	*A.alternata* complex	Cultivated rocket	Bagnolo (TO)
Ruc 3/10	*A.alternata* complex	Cultivated rocket	Alessandria (AL)
Ruc 4/10	*A.alternata* complex	Wild rocket cv. Frastagliata	Albenga (SV)
Ruc 5/10	*A. arborescens*	Cultivated rocket	Moncalieri (TO)
Ruc 6/10	*A.alternata* complex	Cultivated rocket	Moncalieri (TO)
Ruc 9/10	*A. arborescens*	Cultivated rocket	Moncalieri (TO)
Ruc 12/10	*A. arborescens*	Cultivated rocket	Moncalieri (TO)
Ruc 13/10	*A.alternata* complex	Cultivated rocket	Moncalieri (TO)
Ruc 1/11	*A.alternata* complex	Wild rocket	Moncalieri (TO)

**Table 6:** List of the isolates of *Alternaria* spp. obtained from seeds of different hosts.

Isolate Code	Species	Host	Origin
Ruc PMP 4	*A. brassicicola*	Cultivated rocket	Cesena (FC)
Ruc PMP 8	*A.alternata* complex	Cultivated rocket	Cesena (FC)
Ruc PMP 9	*A.alternata* complex	Cultivated rocket	Cesena (FC)
Ruc PMP 12	*A.alternata* complex	Cultivated rocket	Cesena (FC)
Ruc PMP 19	*A. japonica*	Cultivated rocket	Cesena (FC)
36Q-4NL	*A. japonica*	Wild rocket	Venezia (VE)
37Q-16NL	*A. japonica*	Wild rocket	Venezia (VE)
38Q-1NL	*A. japonica*	Wild rocket	Lodi (LO)
38Q-9NL	*A. japonica*	Wild rocket	Lodi (LO)
38Q-19NL	*A. japonica*	Wild rocket	Lodi (LO)
43Q-1NL	*A. japonica*	Wild rocket	Azzano (BG)
43Q-2NL	*A. japonica*	Wild rocket	Azzano (BG)

### 4.2. Secondary Metabolite Production in Vitro

Production of secondary metabolites was tested for each isolate using a modified Czapek-Dox medium according to Brzonkalik *et al.* [28]: 10 g/L glucose, 0.162 g/L NH_4_NO_3_, 1.7 g/L KH_2_PO_4_, 0.85 g/L MgSO_4_, 0.425 g/L NaCl, 0.425 g/L KCl, 0.017 g/L FeSO_4_, 0.017 g/L ZnSO_4_ and 1.7 g/L yeast extract, pH 5.5. Cultures were inoculated with three mycelia plugs in 50 mL of medium. All cultures were performed in triplicate and incubated in the dark at 28 °C. After 8 days, cultures were filtered and the clear medium was analyzed.

### 4.3. Mycotoxin Evaluation on Infected Plants

In order to test *in vivo* production of mycotoxins, 13 isolates of *Alternaria* spp., 8 from rocket and 5 from cabbage, were randomly selected and propagated on PCA as previously described. Forty to fifty day-old plants of cultivated rocket, cauliflower and cabbage grown in 2 L pots were inoculated by spraying a suspension containing conidia and mycelia fragments at 1 × 10^5^ CFU/mL. Inoculated plants were immediately covered with plastic bags for 7 days and kept in a growth chamber at 23 ± 1 °C. Non inoculated plants maintained in the same condition were used as control.

Fifteen days after artificial inoculation, the percentage of affected leaf area of 50 leaves was estimated by using a disease index scale ranging from 0 to 5; where 0 = no visible leaf spots; 1 = up to 10% leaf area affected; 2 = 11%–25% leaf area affected; 3 = 26%–50% leaf area affected; 4 = 51%–75% leaf area affected; 5 = more than 75% or dead leaves [30]. The disease index was converted into percentage. The infected leaves of rocket, cauliflower and cabbage were collected and stored at −20 °C for 15 days before starting the evaluation. Disease reactions were assigned as follows: NP, non-pathogenic strain; L, low virulence (10%–30%); M, moderate virulence (31%–50%); H, high virulence (51%–100%).

### 4.4. Standard Preparation for Chemical Analysis

Standards of TeA copper salt from *A. alternata* (Purity ≥ 98%), AOH from *Alternaria* spp. (Purity ≥ 94%), AME from *Alternaria alternata* (Purity ≥ 98%), ALT from *Alternaria* spp. (Purity ≥ 98%) and TEN from *Alternaria tenuis* (Purity 99%) were purchased from Sigma-Aldrich in crystallized form. A stock solution of 1000 μg/mL and a working solution of 10 μg/mL were prepared in methanol for each molecule and kept at −20 °C. Standards for HPLC calibration and standards for addition experiment were prepared by diluting the working solution.

### 4.5. Extraction of Secondary Metabolites from Fungal Cultures

*Alternaria* mycotoxins were extracted by liquid-liquid extraction. Each sample was adjusted to pH 2 with HCl and an aliquot (5 mL) was transferred in a separating funnel. Ten mL of dichloromethane were added three times and the mixture was shaken for 1 min, then the dichloromethane extracts were collected in a flask. The final extract was evaporated to dryness in a rotary evaporator at 35 °C. The residue was dissolved in 1 mL of H_2_O:CH_3_OH 1:1 for the HPLC-MS/MS analysis.

### 4.6. Extraction of Secondary Metabolites from Plant Material

*Alternaria* mycotoxins were extracted by solid-liquid extraction following a method described by Noser *et al.* [31]. All samples were homogenized and stored in a refrigerator at −20 °C until extraction. 1 g of each homogenized sample was placed in a centrifuge tube with 0.5 g of NaCl and 10 mL of extraction solution (CH_3_OH:CH_3_CN:H_2_O 10:45:45 *v*/*v*/*v* adjusted to pH 3 with o-phosphoric acid) was added. The mixture was shaken for 30 min in an ultrasonic bath and then centrifuged at 5000 rpm for 5 min.

Solid phase extraction was performed using hydrophilic lipophilic balanced (HLB) copolymer cartridges (Polyntell AttractSPETM W/O 3 mL, 60 mg). First, 2 mL of clarified extract was diluted with 18 mL of HCl 0.01 N then passed through a conditioned cartridge (conditioning was made first with 3 mL of CH_3_OH followed by 3 mL of H_2_O). After a washing step with 3 mL of water, toxins were eluted with CH_3_OH:CH_3_CN 70:30 acidified with 0.1% of CH_3_COOH. The eluate was dried in vacuum concentrator (Eppendorf) at 30 °C, and the extract was then dissolved in 1 mL of H_2_O: CH_3_OH 1:1 for the HPLC-MS/MS analysis.

### 4.7. HPLC-MS/MS Analysis of Secondary Metabolites

All analyses were carried out by using a 1260 Agilent Technologies system consisting of a binary pump and a vacuum degasser, connected to a Varian autosampler Model 410 Prostar (Hansen Way, CA, USA) equipped with a 20 μL loop coupled with a Varian 310-MS TQ Mass Spectrometer. The separation of mycotoxins was performed using a Kinetex PFP (100 × 2.10 mm 2.6 μm, Phenomenex, Torrance, CA, USA) under a flow of 200 μL/min. The column temperature was set at 35 °C. Solvent A was H_2_O with 2 mM NH_4_HCO_3_, solvent B was CH_3_OH with 2 mM NH_4_HCO_3_. HPLC analysis was performed using a linear gradient from 40% to 100% of solvent B in 12 min.

Samples were ionized using an electrospray (ESI) ion source operating in negative ion mode. For the MRM experiments two transitions were selected for each compound: for TeA, *m*/*z* 196 > 112 CE 24V (monitoring) and *m*/*z* 196 > 139 CE 20V (quantification); for AME, *m*/*z* 257 > 147 CE 34V (monitoring) and *m*/*z* 257 > 213 CE 22V(quantification); for AOH, *m*/*z* 271>228 CE 28V (monitoring) and *m*/*z* 271 > 256 CE 22V (quantification); for ALT, *m*/*z* 291 > 247 CE 20V (monitoring) and *m*/*z* 291 > 229 CE 12V (quantification); for TEN, *m*/*z* 413 > 141 CE 18V (monitoring) and *m*/*z* 413 > 271 CE 16V (quantification). The collision gas (Ar) pressure was set at 2 mbar for all experiments.

## 5. Conclusions

The spread of *Alternaria* leaf spot on rocket and cabbage represents not only a danger for the production chain but also a serious risk for human health. From our results, *in vitro* assay is a potential tool to predict the possible mycotoxin contamination, except for tenuazonic acid, of these host plants grown in field or under greenhouse conditions.

## Abbreviation

TeA: tenuazonic acid
AOH: alternariol
AME: alternariol monomethyl ether
ALT: altenuene
TEN: tentoxin
